# Isolation of *Zygosaccharomyces siamensis* kiy1 as a novel arabitol-producing yeast and its arabitol production

**DOI:** 10.1186/s13568-023-01581-4

**Published:** 2023-07-15

**Authors:** Kan Iwata, Mayumi Maeda, Yutaka Kashiwagi, Kenji Maehashi, Jun Yoshikawa

**Affiliations:** 1 Department of Fermentation Science and Technology, Graduate School of Applied Bioscience, Tokyo University of Agriculture, 1-1-1 Sakuragaoka, Setagaya-Ku, Tokyo, 156-8502 Japan; 2 Department of Fermentation Science, Faculty of Applied Bioscience, Tokyo University of Agriculture, 1-1-1 Sakuragaoka, Setagaya-Ku, Tokyo, 156-8502 Japan

**Keywords:** Arabitol, Sugar alcohol, *Zygosaccharomyces siamensis*, Honey

## Abstract

Arabitol is gaining attention in the food industry as an alternative sweetener owing to its low-caloric and non-cariogenic characteristics. The yeast strain kiy1 was newly isolated from unpasteurized honey for arabitol production. Based on internal transcribed spacer sequence analysis, the isolated strain was identified as *Zygosaccharomyces siamensis*. In this study, the effects of different substrates and sugar concentrations on arabitol production were investigated. When three types of carbon sources (glycerol, fructose, and glucose) were used, glucose was the most suitable substrate for arabitol production (68.7 g/L). Maximum arabitol production (101.4 g/L) was observed at a glucose concentration of 30%, and the highest arabitol production yield was 0.34 g/g of initial glucose. In the time-course production of sugar alcohols by strain kiy1, glucose was completely consumed for 8 days. The concentration of arabitol exceeded that of glycerol after 3 days, and the final arabitol concentration reached 83.6 g/L after 10 days. The maximum production rate was 16.7 g/L/day. The yeast produced glycerol as an intracellular sugar alcohol in the early stage of culture and switched its metabolism to arabitol production after the middle stage. *Z. siamensis* kiy1 possessed an NADP^+^-dependent arabitol dehydrogenase, which indicated that it probably produces arabitol via ribulose from glucose. These results suggest that the novel yeast strain, *Z. siamensis* kiy1, is promising for arabitol production. The proposed arabitol production approach can contribute toward its production at the industrial scale.

**Graphical Abstract:**

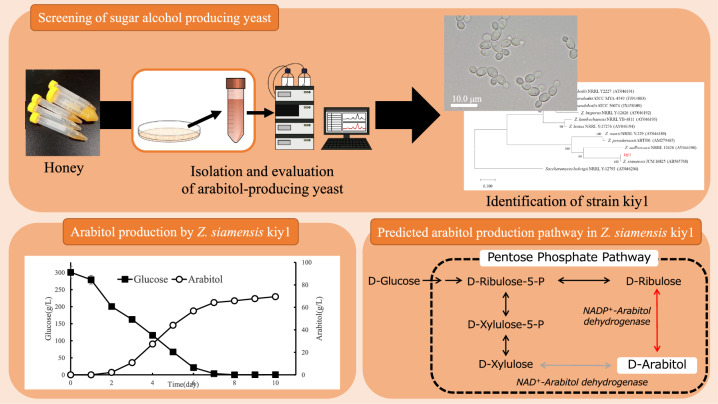

## Key points

- *Z. siamensis* kiy1 was isolated as an arabitol producer from unpasteurized honey.
- *Z. siamensis* kiy1 produces arabitol under the high glucose conditions.
- *Z. siamensis* kiy1 may produce arabitol by NADP-dependent arabitol dehydrogenase.

## Introduction

Sugar alcohols are sugars in which the carbonyl group of an aldose or ketose has been reduced. They are used as alternative sweeteners because they exhibit a mild sweet taste, similar to sucrose, as a common characteristic. They do not undergo Maillard reaction due to the absence of carbonyl groups and have superior resistance to heat, acids, and alkalis in comparison to reducing sugars. Physiologically, they are low-caloric, non-cariogenic, insulin-independent, and do not increase blood glucose levels after ingestion. However, sugar alcohols also exert a transient laxative effect upon overdose (Carocho et al. 2017). Arabitol is a 5-carbon sugar alcohol and an isomer of xylitol and ribitol. In 2004, the U.S. Department of Energy selected polyols, such as xylitol and arabitol, as a type of the 12 useful chemicals for industrial biorefineries (Erickson et al. 2012). It can be produced by laboratory-scale fermentation from glucose and glycerol using the yeasts *Zygosaccharomyces rouxii*, *Debaryomyces*, and *Candida* (Saha et al. 2007; Kumdam et al. 2013; Yoshikawa et al. 2014). Arabitol has significantly higher sweetness to calories ratio than xylitol (Loman et al. 2018). It is relatively low-glycemic which limits carbohydrate ingestion similar to other sugar alcohols. Thus, it has gained attention in the food industry as an alternative sweetener (Koganti et al. 2011). Arabitol can also be converted to xylitol, which is in high demand, using *Gluconobacter oxydans* (Sugiyama et al. 2003).

Arabitol is chemically synthesized by a two-step hydrogenation process using arabinoic acid and lactone as the starting materials (Kumdam et al. 2013). This process uses a rare material and an expensive catalyst, such as Raney nickel, for the reducing reaction. The reaction condition requires high temperatures (over 100 °C) and high pressures in a hydrogen atmosphere. The large number of by-products formed during the process must be removed and purified in a costly and complex procedure (Kumdam et al. 2013). Therefore, biological processes from sustainable biomass using microorganisms are required because they are inexpensive and have a low environmental impact. Although examples of arabitol production by several yeast species have been reported, extensive culture times and insufficient productivity of arabitol are major problems. Therefore, screening osmophilic yeasts with high arabitol yields and optimizing culture conditions are extremely important for the industrial production of arabitol. Moreover, improved understanding of metabolic behavior can reduce the production of by-products and increase the production of target compounds. Thus far, investigation of the metabolism of arabitol production is limited.

In this study, arabitol-producing yeasts were isolated from dried fruits, flowers, fruits, honey, and miso. Among them, the strain kiy1, which was obtained from unpasteurized honey, was identified as *Zygosaccharomyces siamensis*, and its culture conditions for arabitol production were investigated. Furthermore, the metabolic shift toward arabitol production in this yeast strain was demonstrated by quantifying intracellular and extracellular sugar alcohol production and measuring enzyme activities related to arabitol production.

## Materials and methods

### Culture media

The screening medium (200 g/L glycerol, 10 g/L yeast extract, and 5 g/L polypeptone) was used to enrich osmotolerant microbes from the natural samples. The isolation agar medium (200 g/L glycerol, 10 g/L yeast extract, 5 g/L polypeptone, 20 g/L agar, and 50 mg/L chloramphenicol) was used to isolate yeast from the enrichment-culture broths in which sugar alcohol production was confirmed. The basic fermentation medium and pre-culture medium contained 300 g/L and 20 g/L glucose with 10 g/L yeast extract and 5 g/L polypeptone, respectively.

### Screening of sugar alcohol-producing yeasts

The natural samples were inoculated in test tubes containing 5 mL of the screening medium and were cultivated at 30 °C for 7 days with reciprocal shaking at 180 spm. The production of sugar alcohols in the culture medium was confirmed by high-performance liquid chromatography (HPLC) as described below. The culture broth containing sugar alcohols was added to the isolation medium and incubated at 30 °C for 2 days. Arabitol production by the isolated colonies was confirmed using the same method in the screening medium.

### Identification of strain kiy1

Cell pellets of the isolated yeasts on the agar medium were suspended in 10 mM Tris–HCl buffer (pH 8.0) containing 1 mM EDTA and 0.1% zymolyase-20T (Nacalai Tesque, Kyoto, Japan) and incubated at 30 °C for 1 h. After heating at 100 °C for 5 min, the centrifuged supernatant was used as a template for DNA sequence analysis. The internal transcribed spacer (ITS) region of the ribosomal RNA gene was amplified by PCR with KOD FX (Toyobo, Osaka, Japan) using ITS1 and ITS4 primers (Valente et al. 1996). The PCR protocol was performed as follows: after initial denaturation at 94 °C for 2 min, 30 cycles of (1) denaturation at 94 °C for 10 s, (2) annealing at 55 °C for 30 s, and (3) extension at 68 °C for 1 min. DNA sequences were analyzed using the Basic Local Alignment Search Tool (BLAST) program (https://blast.ncbi.nlm.nih.gov/Blast.cgi) against sequences registered in the DNA Data Bank of Japan (DDBJ)/European Nucleotide Archive (ENA)/GenBank database. The obtained sequence (accession no. LC727651) was aligned with the ITS sequences of other *Zygosaccharomyces* spp. type strains using MEGA X, and a molecular phylogenetic tree was constructed with the neighbor joining method using the same program. The ITS sequence of *Saccharomycodes ludwigii* NRRL Y-12793 was used as the outgroup (Hulin and Wheals 2014).

### Analytical methods

One mL of the broth was centrifuged at 10,000 ×*g* for 10 min. The supernatant was diluted and filtered through a membrane filter with a pore size of 0.45 μm. The concentrations of glucose, glycerol, and arabitol in the filtered sample were measured by HPLC with Coregel-87C column (7.8 × 300 mm; Concise Separations, CA, USA) using H_2_O as the mobile phase at flow rate of 0.6 mL/min and column temperature of 80 °C. The peaks were detected with refractive index detector 5450 (Hitachi High-Tech Science, Tokyo, Japan). Sugar alcohol concentrations were determined from standard curves prepared with authentic concentrations.

### Arabitol production by strain kiy1

Cultures for arabitol production were performed in the basic medium with different carbon sources and various concentrations. One loopful of yeast colony on the agar medium was inoculated into a 100 mL-Erlenmeyer flask containing 15 mL of the liquid medium, and then cultured at 30 °C for 9 days with rotary shaking at 200 rpm.

Scale-up culture for arabitol production was performed in 500 mL-Erlenmeyer flasks containing 100 mL of the basic fermentation medium. As pre-culture, one loopful of the colony was inoculated into a 100 mL-Erlenmeyer flask containing 10 mL of the pre-culture medium, and then was cultured at 30 °C for 2 days with rotary shaking at 200 rpm. Next, the pre-culture broth was inoculated into a 500 mL-Erlenmeyer flask with OD_660_ of 0.2, and it was cultured for 9 days under the same conditions. The amount of arabitol in the culture supernatant and yeast growth were sequentially determined using HPLC and OD_660_, respectively.

### Analysis of intracellular sugar alcohol

After cultivation, the yeast cells were washed with saline and suspended in 50 mM Tris–HCl buffer (pH 7.2) with glass beads. Next, the suspension was vigorously mixed at 4 °C for 1 h. The amounts of arabitol and glycerol in the supernatant after centrifugation at 3300 ×*g* for 5 min were determined by HPLC.

### Enzyme activity of ArDH

The broths at days 1 and 4 were centrifuged at 3300 ×*g* for 5 min, and the yeast cells were washed with saline. After it was crushed under the same conditions as described in the previous section, the suspension was centrifuged at 12,000 ×*g* for 20 min at 4 °C, and the supernatant was used as the crude enzyme solution. One mL of the enzyme reaction mixture contained 0.1 mL of crude enzyme solution, 20 μmol of arabitol, 1.0 μmol of NAD^+^ or NADP^+^, and 50 μmol of Tris–HCl buffer (pH 9.0). Enzymatic reactions were performed using an absorbance spectrometer maintained at 30 °C. The reaction was initiated following substrate addition, and the time-dependent increase in absorbance at 340 nm based on NAD^+^/NADP^+^  reduction was measured. One unit of the enzyme activity was defined as the amount of enzyme that produced 1 nmol of NADH/NADPH per min. The molecular absorption coefficient of NADH/NADPH was 6.22 × 10^3^ (mM^−1^ cm^−1^) (Wong et al. 1993; Ingram and Wood 1965). Protein amounts were determined by the Bradford method using bovine serum albumin as the standard protein.

## Results

### Screening and isolation of arabitol-producing yeast

Thirty-five samples (dried fruit, flower, fruit, honey, and miso) were inoculated in the screening medium and grown in enrichment culture. A total of 170 colonies were isolated on isolation agar medium: 8 from 2 types of dried fruits, 30 from 11 types of flowers, 18 from 3 types of fruits, 79 from 5 types of honey, and 35 from 14 types of miso. Among them, 9 strains from honey produced arabitol from glycerol and no other sugar alcohols.

### Identification of strain kiy1

**Fig. 1:**
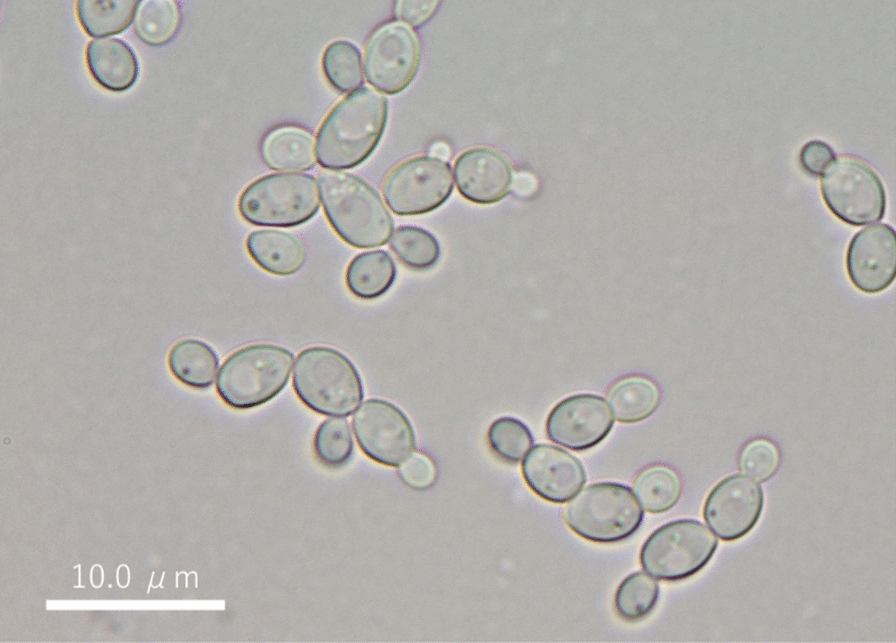
Micrograph of strain kiy1. The bar length represents 10 μm

**Fig. 2:**
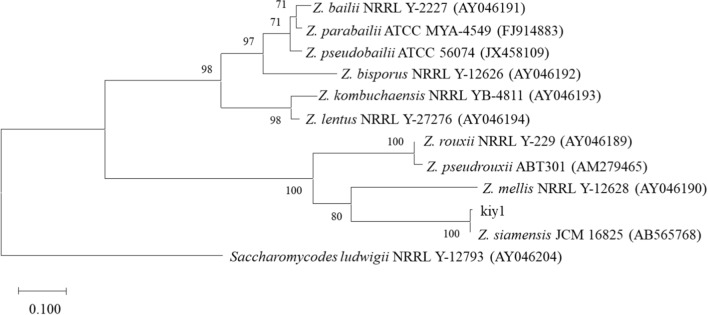
Phylogenetic tree of strain kiy1 based on the internal transcribed spacer region DNA sequence. The numbers in parentheses indicate the accession number. The numbers at node show bootstrap values. The scale bar represents sequence divergence

Homology analysis of the ITS region sequences in the rDNA of 9 isolates which produced arabitol were performed. Seven strains from unpasteurized honey (kiy1 to 7) and two strains from pasteurized honey (kig1 and 2) were closely related to *Zygosaccharomyces siamensis* and *Candida magnoliae*, respectively. A micrograph of strain kiy1, the most arabitol producer of 9 isolates, was shown in Fig. 1. Molecular phylogenetic tree analysis was performed using the ITS sequences of strain kiy1 and type strains of the genus *Zygosaccharomyces* divided into two large clades (Fig. 2). Strain kiy1 was categorized in a small clade with strain JCM 16825 and, together with the homology (99.32%) in the BLAST analysis, was identified as *Z. siamensis*. The strain was deposited as NBRC 116063 in the NITE biological resource center (NBRC, Chiba, Japan).

### Arabitol production by *Z. siamensis* kiy1

**Fig. 3:**
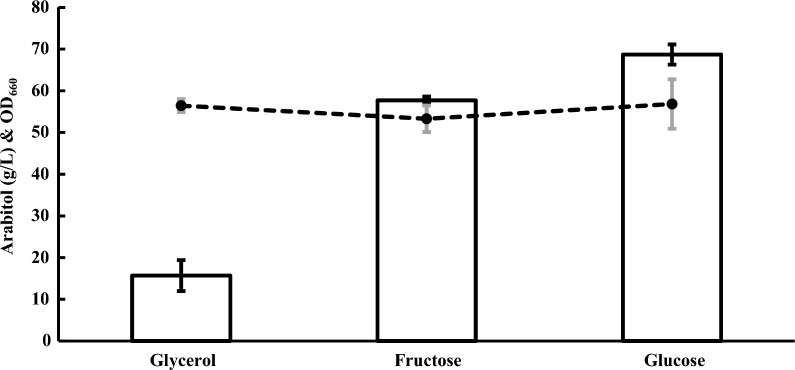
Arabitol production by *Z. siamensis* kiy1 with different carbon sources. White bar and closed circle represent the amount of arabitol and OD_660_, respectively. The data show the means ± standard deviations of three independent experiments

Arabitol production by *Z. siamensis* kiy1 was performed using glucose, fructose, or glycerol as a carbon source in the screening medium (Fig. 3). There was no difference in the growth of yeast in the whole media. Arabitol production was 15.7 g/L in the glycerol medium, which was the carbon source for screening. Large amounts of arabitol (57.7 and 68.7 g/L) were produced in the fructose and glucose media, respectively.

**Fig. 4:**
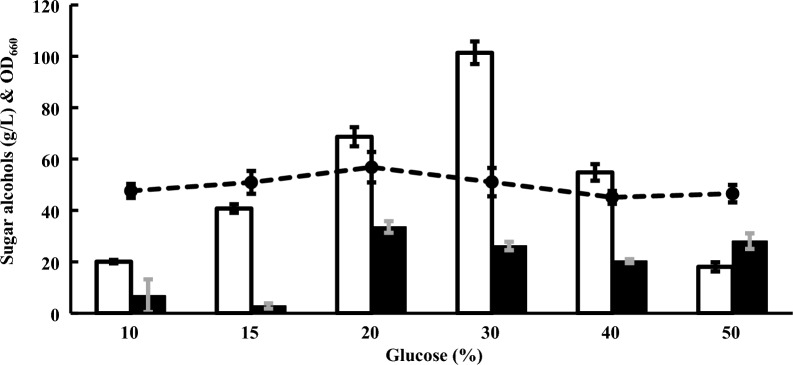
Arabitol production by *Z. siamensis* kiy1 with different glucose concentrations. White bar, black bar, and closed circle represent the amount of arabitol, amount of glycerol, and OD_660_, respectively. The data show the means ± standard deviations of three independent experiments

This yeast was cultivated with various concentrations of glucose, which was the carbon source with the highest arabitol production (Fig. 4). The maximum arabitol production was 101.4 g/L from 30% glucose, and 26.2 g/L glycerol was also produced. Higher sugar concentrations of 40 and 50% glucose were not fully consumed after 9 days of incubation. Arabitol production under both conditions was substantially decreased, but glycerol was produced over 20 g/L.

### Time course of sugar alcohol production by *Z. siamensis* kiy1

**Fig. 5:**
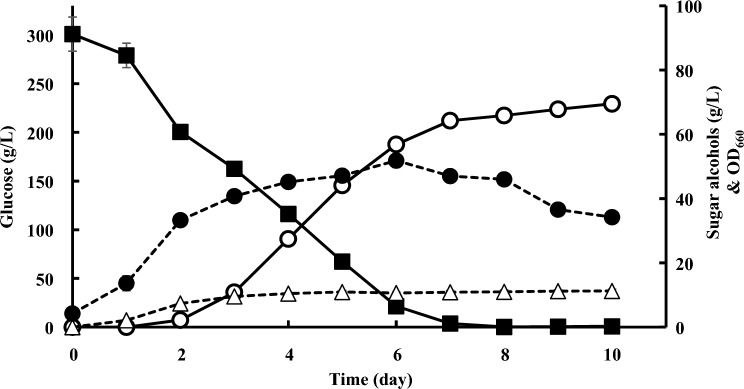
Time course of arabitol production by *Z. siamensis* kiy1. Closed square, open circle, open triangle, closed circle represent the amount of glucose (■), amount of arabitol (〇), amount of glycerol (△), and OD_660_ (●), respectively. The data show the means ± standard deviations of three independent experiments

To examine the production rate of arabitol by *Z. siamensis* kiy1, the time course of arabitol production in the basic fermentation medium was observed for 10 days (Fig. 5). Glucose was completely consumed after 8 days. Arabitol was detected after 2 days, and its concentration was higher than that of glycerol after 3 days. The final arabitol concentration reached 83.6 g/L after 10 days. Glycerol production was confirmed after 1 day, and the concentration was maintained at approximately 10 g/L after 4 days. Yeast growth appeared to enter a stationary phase after 4 days, with OD_660_ of over 50. The total production yield to consumed glucose was 0.23 g/g-glucose, and the maximum production rate was 16.7 g/L/day at 4-day cultivation.

**Fig. 6:**
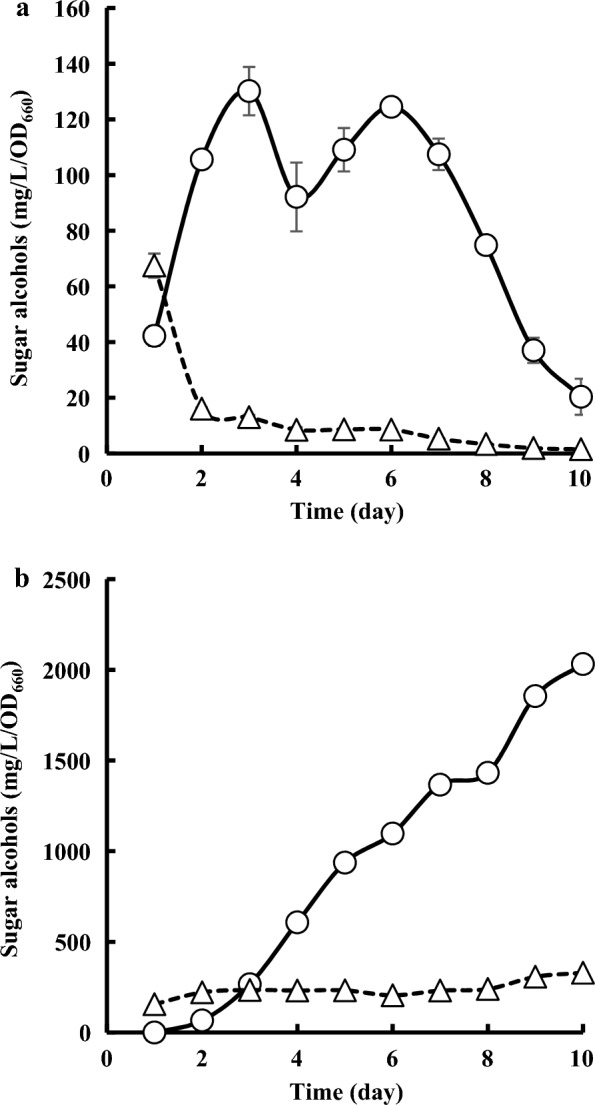
Intracellular (**a**) and extracellular (**b**) sugar alcohol production to OD_660_ by *Z. siamensis* kiy1. Open circle and open triangle represent the amount of arabitol (〇) and glycerol (△), respectively. The data show the means ± standard deviations of three independent experiments

In addition, intracellular and extracellular sugar alcohol productions at OD_660_ were analyzed (Fig. 6a and b). Intracellular sugar alcohol production was observed after 1 day, and the concentration of glycerol at OD_660_ was higher than that of arabitol. Glycerol production then decreased, and the concentration of arabitol became higher than that of glycerol after 2 days. Extracellular arabitol was accumulated more than glycerol after 4 days in proportion to intracellular arabitol production. Glycerol accumulation did not increase after 2 days, owing to low intracellular production.

### Enzyme activity of arabitol dehydrogenase (ArDH)

Cell-free extracts from *Z. siamensis* kiy1 cultivated for 1 and 4 days were prepared, and the ArDH activities were assayed as 4.15 and 15.0 U/mg-protein, respectively (Table 1). The enzyme activity at the 4-day cultivation (the arabitol-production phase) tended to be higher than that at the 1-day cultivation (the glycerol-predominant phase). The activity of ArDH was only confirmed when NADP^+^ was added, and NAD ^+^ was not used as a cofactor.

**Table 1:** Enzyme activity of arabitol dehydrogenase (ArDH). The data show the means ± standard deviations of three independent experiments

Cultivation Time	ArDH activity (U / mg-protein)	
	NAD^+^	NADP^+^
1 day	N.D	4.15 ± 2.62
4 days	N.D	15.0 ± 7.91

*ND* not detectable

## Discussion

In this study, a novel yeast strain, *Zygosaccharomyces siamensis* kiy1, was isolated from unpasteurized honey and produced arabitol from glycerol, fructose, and glucose. *Z. siamensis* is a relatively new species that was isolated from honey in 2011 (Saksinchai et al. 2012). In physiological studies, phenotypic differences from the closely related *Z. mellis* were observed in the assimilation of galactose and erythritol, growth at 37 °C, and growth in the presence of salt. In a simplified approach, discrimination was achieved by comparing the sequences of the 26S rDNA D1/D2 domain and ITS region and by forming the ascospore. The species name “siamensis” was designated from Siam, the historical name of Thailand, in which the yeast was isolated. There have been no reports of sugar alcohol production by *Z. siamensis*. Although arabitol was not detected in honey (data not shown), its production by *Z. siamensis* may be a defense mechanism in a hyperosmotic environment. It is generally known that yeasts produce sugar alcohols to regulate osmotic pressure inside and outside the cell (Hohmann. 2002).

*Z. siamensis* kiy1 produced the most arabitol when glucose was used as the carbon source, and the highest arabitol production yield was 0.34 g/g to 30% glucose (Fig. 4). Although the yields of arabitol cannot be simply compared due to differences in culture conditions, those by other yeasts, *Hansenula polymorph*a DSM 70277 (Escalante et al. 1990), *Metschnikowia reukaufii* AJ14787 (Nozaki et al. 2003), *Zygosaccharomyces rouxii* NRRL Y-27624 (Saha et al. 2007), *Kodamaea ohmeri* NH-9 (Zhu et al. 2010), *Pichia anomala* TIB- × 229 (Zhang et al. 2014), and *C. parapsilosis* SK26.002 A6 (Zheng et al. 2020) were 0.14 g/g, 0.52 g/g, 0.48 g/g, 0.41 g/g, 0.22 g/g, and 0.27 g/g, respectively. In fed-batch culture, *Z. rouxii* ZR-5A produced 149.10 g/L of arabitol for 144 h using glucose as the carbon source with a productivity of 1.04 g/L/h (Li et al. 2023). Arabitol production of *Wickerhamomyces anomalus* WC 1501 reached 265 g/L for 325 h with a yield of 0.74 g/g and productivity of 0.82 g/L/h in fed-batch culture using glycerol as carbon source (Raimondi et al. 2022). In *Yarrowia lipolytica* ARA9, D-arabitol production reached 118.5 g/L in 108 h with a yield of 0.49 g/g and productivity of 1.10 g/L/h in fed-batch culture using glycerol (Yang et al. 2021). Several yeasts produced higher yields than that with *Z. siamensis* kiy1, but they were cultivated in media with less than or equal to 20% glucose as the carbon source. Thus, the arabitol yield of strain kiy1 under the higher glucose condition (30%) was remarkable, and the productivity may be increased by fed-batch culture.

As shown in Fig. 6a, the intracellular production of sugar alcohols in *Z. siamensis* kiy1 predicted that the strain would produce glycerol to regulate osmotic pressure in the early stage of culture, and would switch the metabolism to arabitol production with the related enzyme expression after the middle stage. The temporary decrease in intracellular arabitol at day 4 may be caused by extracellular release of arabitol because the slope of extracellular arabitol levels was steeper (Fig. 6b).

**Fig. 7:**
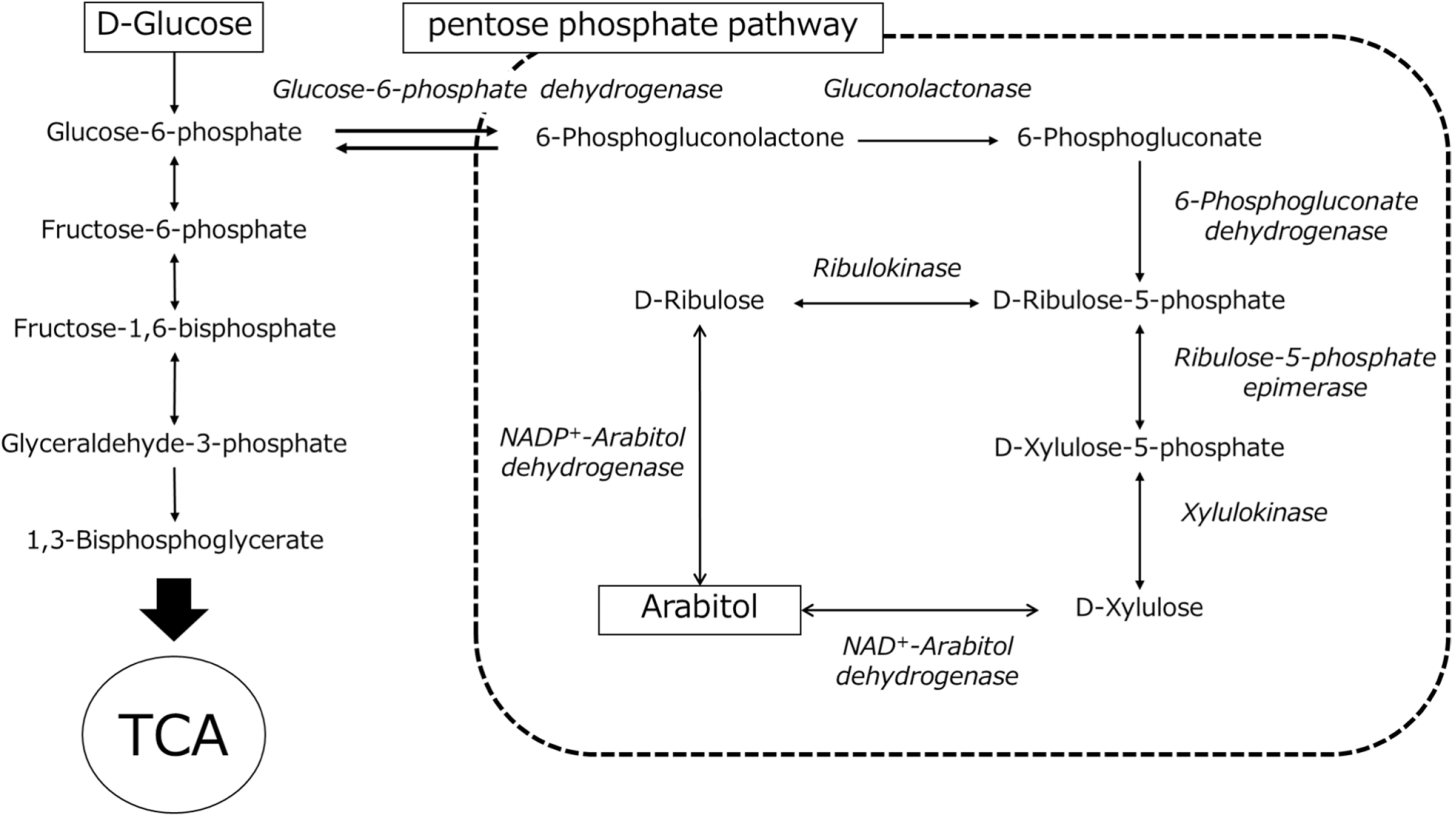
Metabolic pathway involved in arabitol production in yeast. The pathway is proposed based on the report by Qi et al. (2015) and Zheng et al. (2020)

Arabitol production by yeast has been reported to occur through two alternative pathways from glucose via the pentose phosphate pathway (Qi et al. 2015; Zheng et al. 2020). After glucose is phosphorylated to glucose-6-phosphate, it is converted to D-ribulose-5-phosphate. D-ribulose-5-phosphate is dephosphorylated by ribulokinase and D-ribulose is reduced to arabitol by NADP^+^-dependent arabitol dehydrogenase (ArDH). In another pathway, D-ribulose-5-phosphate is converted to D-xylulose-5-phosphate by ribulose-5-phosphate epimerase, and after dephosphorylation by xylulokinase, D-xylulose is reduced to arabitol by NAD^+^-dependent ArDH (Fig. 7). In this study, NADP^+^ was used only as a coenzyme when ArDH activity in *Z. siamensis* kiy1 was measured (Table 1). This result suggests that the yeast has the pathway to produce arabitol from ribulose. *Z. rouxii* had two ArDHs because the activity was measured under both NAD^+^ and NADP^+^ (Ingram and Wood 1965). However, NADP^+^-dependent activity was significantly higher. In contrast, *Candida albicans* was reported to have only NAD^+^-dependent ArDH (Wong et al. 1993). Because ArDH is the final step in arabitol production, enzyme activity may directly influence arabitol yield.

In this study, a novel arabitol-producing yeast, *Zygosaccharomyces siamensis* kiy1, was isolated, and a portion of its arabitol production metabolism was evaluated by measuring intracellular sugar alcohol production and ArDH activity. In the future, it may be possible to obtain a high arabitol-producing mutant by overexpressing ArDH. It may also be possible to elucidate the physiological role of arabitol in arabitol-producing yeasts by deleting this enzyme. Bioengineering approaches are expected to further progress arabitol production.

## Data Availability

The datasets used and/or analysed during the current study are available from the corresponding author on reasonable request.
